# Relocate to compete: a critical view on the diaspora of Russian athletes

**DOI:** 10.3389/fspor.2025.1603414

**Published:** 2025-07-02

**Authors:** Timur Absalyamov, Mathias Schubert

**Affiliations:** ^1^ Independent Researcher, Kazan, Russia; ^2^ Institute of Sport Science, Johannes Gutenberg University Mainz, Mainz, Germany

**Keywords:** sport migration, sporting nationality, sanctions, Russia, athlete rights

## Abstract

Among the many areas experiencing the growing interplay between geopolitical dynamics and sport is athlete migration and sporting nationality change. These often occur as a reaction to factors such as economic incentives, career prospects, political stability, or personal safety. The current scholarship on this topic is, however, largely underdeveloped. This brief research report critically assesses data on the migration of Russian athletes following the 2022 conflict in Ukraine and related sanctions on Russia. The report reveals that the number of Russian athletes who changed their sporting nationality in order to compete continues to rise, with key sports affected at the moment being chess, figure skating, wrestling, equestrian, auto racing, and soccer. This wave of migration includes both top-level and mid-level athletes, notably featuring six medalists from the 2024 Olympics. The destinations chosen by Russian athletes post-2022 largely align with broader Russian migration patterns, while some also relocated to sporting powerhouses in the Global North. Perspectives on this migration vary depending on stakeholders: state officials stress the investments made in athlete development and call for compensation or loyalty, whereas fellow athletes frame migration as an individual necessity dictated by career prospects and longevity. Athlete migration highlights unique challenges in governance, ethics, and policy-making within sports. Based on our case study, future research directions are outlined to empirically examine the situation through the lens of sports ethics and integrity.

## 1 Introduction

### 1.1 Context and study objectives

Athlete migration and changes in sporting nationality have become increasingly prominent in the modern sporting landscape. Driven by factors such as financial incentives, career advancement, political stability, and personal security, athletes began to frequently seek opportunities beyond their original country. As this phenomenon continues to expand, scholarly interest in the subject has grown accordingly (1). While some nationality changes stem from personal circumstances, such as marriage or a desire to represent one's ancestral homeland, researchers have also examined systematic patterns of strategic naturalisation (e.g., Qatar's recruitment of footballers ahead of the 2022 FIFA World Cup) (2).

Beyond deliberate sporting strategies, mass relocation of athletes is often a consequence of broader geopolitical upheavals that trigger large-scale migration (1). A striking example is the displacement caused by the wars in the Balkans during the 1990s, which forced many families from former Yugoslavia to flee their homes. Today, numerous athletes whose parents were part of this migration wave represent their adopted countries on the international stage (3). However, such cases typically involve second-generation athletes rather than active competitors at the time of displacement.

For elite athletes who have actively changed their sporting nationality, the most notable historical examples stem from geopolitical transformations such as the dissolution, unification, or emergence of new states—particularly in the wake of the collapse of communist regimes (4). These developments, combined with the increasingly fast-paced and globalised nature of modern sport, have led some scholars to critically reassess the concept of sporting nationality itself and the broader implications of national allegiance in sport (5, 6).

This brief research report critically assesses a very recent case of athlete migration: the relocation of Russian athletes following the 2022 conflict in Ukraine and related sanctions on Russia. The current scholarship on this topic is still largely underdeveloped. Based on this, the aim of this report is to provide the first descriptive insights into the developments since 2022.

It will be demonstrated in what way athlete migration highlights unique challenges in governance, ethics, and policy-making within sports. For this, the study relies on the theoretical framework of sport integrity. The concept of sport integrity is multifaceted and includes a wide spectrum of issues that distort the sport industry. Gardiner, Parry and Robinson introduced a framework identifying four core dimensions of integrity:

(1) Inherent integrity of sport, referring to the fostering and promotion of virtues within sport (e.g., respect, justice);
(2) Personal integrity, referring to the safeguarding of people participating in sports (e.g., athletes);
(3) Organisational integrity, referring to sport governing bodies’ governance and sustainability;
(4) Procedural integrity, referring to the trustworthiness of sporting competitions (7).

Recently, Manoli and Konstantopoulos proposed to also add a dimension of stakeholder integrity, pointing to the relationship between actors through the lens of a principal-agent logic (8).

Acting through this integrity lens, our insights will inform the drafting of a future research agenda to provide more in-depths and empirical investigations of the topic.

### 1.2 Historic background

In recent years, the global sports landscape has witnessed a growing interplay between geopolitical dynamics and athlete migration, as athletes face complex decisions influenced by international conflicts, sanctions, national talent acquisition policies, and shifting societal norms. These movements not only reflect broader trends in global migration but also highlight unique challenges in governance, ethics, and policy-making within sports.

In 2022, the Russia-Ukraine conflict completely changed the realities of life across the entire post-Soviet region, introducing an entirely new socio-political and economic landscape. Shortly after the conflict began, Russia came under a record number of international sanctions, both from other states and international organisations, which affected many aspects of people's lives (9). As a consequence of these political and economic transformations, estimates from various media sources indicate that between 600,000 and 1.3 million individuals left Russia in 2022–2023 (10). Pinpointing the exact scale of this migration remains challenging, as departures were often spontaneous, and migration patterns proved highly fluid. The main destinations for emigration were either countries where Russian citizens do not need a visa and can communicate in Russian (such as Georgia, Kazakhstan, and Armenia), other visa-free and affordable countries (e.g., Serbia or Turkey) or countries offering favourable conditions for relocated businesses (Cyprus or the UAE) (10).

While a considerable number of emigrants departed Russia spontaneously and without a long-term plan, a significant portion were relocated by their employers (11, 12). Due to the sanctions imposed on Russia, many international company employees were compelled to relocate to other countries. This was due to the fact that financial transactions with Russia became unfeasible, and foreign companies were compelled to leave the Russian market (11). Similarly, some Russian businesses responded to the shifting economic and geopolitical landscape by establishing offices in other countries, enabling them to maintain operations under new constraints, and facilitated the relocation of numerous employees (11).

While the extent to which those who left Russia view their departure as permanent emigration or a temporary move remains uncertain, the term *relocation* (*relokatsiya*) has gained widespread usage in place of *emigration*, as it more precisely captures the nuanced and often temporary nature of these migration processes (13). While some may intend to settle abroad indefinitely, others seem to await the resolution of the conflict before deciding their long-term future: researchers from RANEPA University argue that at least 10% of relocants have returned to Russia, while a total estimate can range anywhere between 15% and 50% (14). The complex motivations driving these migration patterns, ranging from political considerations to the need to circumvent sanction-related work restrictions, remain an area for further academic inquiry. Nevertheless, it is evident that economic sanctions and the international isolation of Russian organisations have significantly hindered many professionals from fully pursuing their careers, ultimately compelling them to relocate.

The case of athletes, however, is more complicated. Unlike other professionals, athletes are tied to national allegiance, which attributes a particular athlete to a particular country. Therefore, when sports governing bodies sanctioned Russia and banned Russian athletes from international competitions, athletes had to change their sporting nationality, not their residency, in order to compete internationally during the sanctions regime. This change introduces a whole new dimension compared to other professions. The Russian case also differs from those illustrated above. Unlike previous instances, it involves established athletes making the conscious decision to change their national affiliation in response to changed conditions.

### 1.3 Academic debate

The body of literature on Russia and the international sanctions imposed in 2022 is expanding rapidly, with a growing number of studies examining the impact of sanctions on Russian sport and the competition bans enforced by global sport governing bodies. Scholars have critically assessed the challenges within international sports governance and debated the application of key principles such as neutrality and sport autonomy in the current geopolitical climate (15, 16). Additionally, researchers have raised concerns about the human rights implications of competition bans on individual athletes (17, 18) and explored the specific repercussions of sanctions on particular sports (19). Nevertheless, broader effects of sanctions, such as subsequent athlete migration, have not been studied yet. Similarly, research on Russian migration triggered by the conflict in Ukraine is still in its early stages. Existing studies primarily focus on documenting the initial phases of this migration wave (20, 21) or analyzing the formation of new Russian emigrant communities (22, 23). However, migration patterns within specific professional groups, such as IT specialists or athletes, have yet to be systematically examined. Thus, the exploration of the Russian athletes’ case closes an important gap in the areas of studies on sporting nationality, the 2022 international sanctions, and Russian migration.

The structure of this brief research report is designed to guide the reader through a comprehensive description of the phenomenon. This forms the basis for the development of a future research agenda. Following the introduction and methods sections, the authors outline the sporting sanctions and competition bans, providing essential context and illustrating the scale of changes affecting Russian athletes. This foundation is followed by an analysis of (a) the sports most impacted by athlete migration and the underlying reasons for this trend; (b) examples of notable athletes that left Russia and their success at the 2024 Summer Olympic Games; (c) the countries that have become popular destinations for Russian athletes; and (d) the typical reactions by Russian authorities and renown athletes. Based on this, the discussion section presents a clear research agenda based on this initial descriptive work.

## 2 Method

This report adopts a case study approach to explore the migration of Russian athletes, enabling detailed examination of individual instances while at the same time identifying broader trends and contextual factors. The analysis is based on a diverse range of secondary sources, including peer-reviewed academic literature, official statements and documents from sports organisations, relevant regulatory texts, and coverage from reputable sports news and analytical portals, which are deemed reliable sources for social science research (24). News articles were sourced via academic databases such as ProQuest, alongside curated aggregators including Google News and Apple News.

To ensure methodological rigour, we applied specific inclusion criteria for source selection. Only sources that demonstrated consistent factual accuracy over time (e.g., established sports news portals with editorial oversight) were considered.

Each case included in the study was substantiated through a cross-referencing process involving independent and unrelated sources. This approach served as a sufficiency measure, ensuring that key facts such as the timing of nationality changes, public announcements, or competition participation, were consistently verified. The description bias—inaccuracies or personal estimates influenced by reporters’ political views—was addressed by cross-referencing multiple sources for each case and focusing exclusively on ‘hard facts’. By ‘hard facts’ in this study we understand verifiable and publicly documented claims, without any subjective interpretation. These include, for example, confirmed changes in citizenship or federation affiliation, registration with new national teams, and formal participation in international competitions. By contrast, speculative commentary, subjective interpretations (e.g., presumed intentions) were excluded from the analysis to maintain analytical rigor.

## 3 The case of Russian athletes

### 3.1 The 2022 sanctions on Russian sport

The international response to Russian sports following the escalation of the Russia-Ukraine conflict in 2022 significantly reshaped the global sports landscape. In the wake of the conflict, numerous international sports federations and governing bodies imposed a series of sanctions designed to isolate Russian sport from the global arena. These measures included the prohibition of Russian athletes, teams, and officials from competing, the removal of national symbols from competition settings, and the relocation or cancellation of events scheduled to take place within Russia (25).

In team sports such as football or basketball, Russian clubs were suspended from international competitions (e.g., UEFA Champions League) and the national teams were similarly banned (25). Moreover, FIFA permitted international players at Russian clubs to suspend their contracts and leave without compensation (19). While sanctions in team sports primarily affected national teams and clubs—thus allowing individual players to compete freely for clubs in other countries—the situation in individual sports was different. In some individual disciplines, where an athlete's national allegiance plays a less central role, such as tennis or mixed martial arts, the restrictions were softer, permitting athletes with Russian nationality to compete at international level (26). However, for most of individual sports there is no particular difference between a national team and an athlete from a particular country, in other words—all the individual athletes, representing a country in international competitions, are eventually considered equal to the national team. Consequently, Russian athletes were effectively banned from competing internationally unless they changed their sporting nationality.

Later in 2023, some sports governing bodies began permitting Russian athletes to compete as ‘neutral’ athletes, albeit under certain eligibility conditions and with vague criteria for the verification (27). All in all, given the short life-span of an athletic career, the Olympic Games happening only once in four years, and uncertainty regarding the future developments, many athletes faced a critical choice: either to change their nationality in order to preserve their prime career opportunities and compete internationally, or to limit themselves to domestic competitions until the situation is resolved.

### 3.2 Sport disciplines

In September 2023, Alexey Morozov, the Deputy Minister of Sport of the Russian Federation, stated that 55 Russian athletes competing in Olympic disciplines had changed their citizenship; when combined with athletes in non-Olympic sports, the total number exceeded one hundred (28). Despite the absence of further official announcements, the most recent estimates for July 2024 indicate that 353 Russian athletes have changed their sporting nationality since February 2022, including 16 world champions, 30 European champions, 32 Russian champions, and numerous medal winners (29). Table 1 provides a breakdown of the migrated athletes amount by sport discipline. Moreover, as sporting nationality transition is a time-consuming and bureaucratic process, the numbers likely reflect the actual picture of migration with a time lag. Thus, by 2025, the number of migrated athletes has likely increased.

**Table 1:** Russian athletes who changed their sporting nationality after February 2022, by sport.

Sport	Athletes	Sport	Athletes
Chess	208	Cycling	4
Figure skating	30	Swimming	4
Equestrian sports	23	Biathlon	3
Martial arts/wrestling	19	Fencing	3
Auto racing	13	Alpine skiing	2
Tennis	8	Motorcycle racing	2
Football (Soccer)	6	Sailing	2
Rhythmic gymnastics	6	Diving	2
Rowing	5	Ice hockey	2
Speed skating	5	Volleyball	1
Athletics	5		

Estimate of Novaya Gazeta, July 2024 (29).

#### 3.2.1 Chess

Notably, the vast majority of migrated athletes are chess players. This disproportionately high number can be explained by the fact that, in 2023, the Russian Chess Federation transferred its affiliation from the European Chess Union to the Asian Chess Federation in order to participate in Asian international competitions during its isolation from Europe. Consequently, the International Chess Federation (FIDE) granted Russian chess players the opportunity to adopt the flag of any European country within a single day, in contrast to the typically lengthy bureaucratic procedures and the need to obtain permission from their original federation (30). Interestingly, discussions about switching the affiliations of Russian national federations from Europe to Asia were active in many sports—especially in football—but aside from chess, only Taekwondo made this move.

#### 3.2.2 Figure skating, gymnastics and wrestling

The second place is taken by figure skaters. Although Russia has always produced a very high number of top figure skaters—and domestic competitions were sometimes considered more competitive than international ones—the phenomenon of “talent export” (i.e., athletes who do not qualify for the national team becoming naturalised in other countries, e.g., Brazilian footballers) was not prevalent prior to 2022 (31). However, following the international competition ban, the number of emigrant athletes has spiked, as the career span in figure skating is often very short, making it crucial for many to seize opportunities immediately. Although the very top figure skaters remained in Russia, a number of regular contenders switched their national allegiance. The most notable example are 2022 Beijing Olympians and the 2021–2022 Russian championship silver medalists in ice dance, Diana Davis and Gleb Smolkin (31).

Similarly, rhythmic gymnastics and wrestling have seen intense domestic competition and a concentration of elite talent. In wrestling, where at Olympics each country fields only one athlete per weight category, earning a national team spot is exceptionally difficult. As a result, many wrestlers have long pursued naturalisation, with Russian-born athletes winning over one hundred medals for other countries at the Olympics and World Championships over the past 30 years (32). Russia's current suspension has naturally accelerated this trend.

#### 3.2.3 Equestrian

Although in Equestrian Russia has not been a prominent power in the 21st century, a big number of leading Russian equestrian athletes also changed their sporting nationality (33). High numbers in this sport can be explained by the fact that it is common for equestrian athletes to live and train abroad, and while being members of the Russian national team, they were already residing elsewhere for many years. Moreover, geopolitical sanctions imposed on Russia prohibit transportation of horses into the country, which is another huge constraint for the equestrian athletes (33). This is reflected in the comment of coach Vladimir Beletsky, who explained the sporting migration of athlete Vladimir Tuganov: “His base and his horses are located outside Russia. Due to sanctions, Tuganov is unable to continue his sporting activities in his homeland because the import of horses into the Russian Federation is prohibited […] With only a few years left in his athletic career, he wants to begin competing now and attempt to qualify for his third Olympics” (34).

#### 3.2.4 Football

An interesting situation occurred in Russian football. Russian players of ethnic origin related to other states are increasingly choosing to represent respective national teams. Ethnic Georgians Saba Sazonov and Konstantin Maradishvili, along with ethnic Armenians Nair Tiknizyan, Eduard Bagrintsev, Georgi Arutyunyan, and Edgar Sevikyan—all born and raised in Russia, and all but Sazonov having played for Russian youth national teams—have switched their sporting nationality to their respective countries since 2022 (35). In an interview, Tiknizyan emphasised the influence of the competition ban: “I couldn’t pass up the chance to play against top national teams on the international stage. Fortunately, I have that opportunity—it was given to me by Armenia, and I'm grateful for it” (36).

All in all, the breakdown by disciplines demonstrates that the numbers are influenced by specific contexts of different sports, and important factors can include particular governance decisions, prevalence of certain sports among particular ethnicities, level of Russian athletes, and practical aspects of every discipline.

We can see that migration patterns largely depend on the decisions of particular sport federations, which raises questions about organisational integrity. For example, the decision to simplify the nationality change process by the International Chess Federation on one hand allowed a number of athletes to compete. On the other, it directly affected the work of the Chess Federation of Russia, literally making a call for athletes to leave it. Further, questions can be raised as to how blanket bans on all Russian athletes imposed by some sport governing bodies can be brought in line with sport's neutrality principle. Different approaches employed by sport governing bodies seem to indicate the necessity for a structured research through the integrity lens.

The migration patterns also hint at broader questions. For example, as noted above, even before the sanctions Russian wrestlers often had to switch nationalities to compete at Olympics, due to quotas defined not on a merit, but on a national basis. How can such a system be viewed in terms of an inherent integrity of sport and procedural (competition) integrity, knowing that it artificially limits the participation of the best athletes in a discipline? Could there be a case to abandon the national allegiance concept at all? Some research on sport and nationalism has already been making such calls in order to improve the quality of competitions and get rid of negative nationalism-related spillovers (37, 38).

Considering existing typologies of athletic migrants, Russian athletes do not fall into any of the existing frameworks: neither do they represent Maguire's *pioneers, settlers, mercenaries, returnees, or nomadic cosmopolitans*; nor are they *transnational settlers, sojourners and mobiles* of Agergaard et al. (39, 40). The closest type of athlete-migrants is *exile* described by Magee and Sugden, who for “…personal or political reasons (either voluntarily or through domestic threats to his career, his liberty, or his life), opts to leave his country of origin to play abroad» [(41), p. 432]. However, it is still far from a perfect fit as Magee and Sugden talk about athletes who perform at other countries, but still represent their own (like many players in team sports). In our case, the key element is the nationality itself. Moreover, the career threats for Russian athletes come at the level of international competitions, and domestically they can keep competing like before, having all the conditions and the infrastructure available. For instance, some of the footballers named above of Georgian and Armenian origin keep playing in the Russian Premier League after the nationality change. This distinction underscores the need to refine existing typologies of athletic migration to account for cases where geopolitical constraints directly impact an athlete's national identity rather than just their place of work. The case of Russian athletes highlights a unique form of migration driven by structural exclusions from international competition, forcing them to navigate between sporting nationality changes and career continuity.

### 3.3 Star athletes

Among the migrated athletes, some acknowledged leaders in their disciplines left Russia. The most famous athletes along with their achievements prior to the nationality change are presented in Table 2 (42–48).

**Table 2:** Star athletes from Russia who changed their sporting nationality (42–48).

Athlete	Sport	Achievements	New country
Alexandra Kosteniuk	Chess	World Champion	Switzerland
Anastasia Kirpichnikova	Swimming	Triple medalist at European Championships	France
Sofya Prosvirnova	Short Track Speed Skating	Five-time European Champion, Three-time Olympian	Denmark
Anna Prakaten	Rowing	Tokyo Olympic silver medalist (single sculls)	Uzbekistan
Alexander Komarov	Greco-Roman Wrestling	Two-time European Championship bronze medalist	Serbia
Mikhail Yakovlev	Cycling	Medalist at World and European Championships	Israel
Chermen Valiev	Wrestling	European Junior Champion	Albania
Dauren Kurugliev	Freestyle Wrestling	European Games & World Cup Champion	Greece
Artur Beterbiev	Boxing (Light-Heavyweight)	World Champion	Canada
Dmitry Bivol	Boxing (Light-Heavyweight)	World Champion	Kyrgyzstan

For the 2024 Paris Olympics, unofficial estimates suggest that 80 athletes born in Russia or USSR, who previously held Russian sporting nationality, participated in the Games (32). In contrast, only 15 athletes with current Russian nationality competed under neutral status (32). Among those 80 athletes, 26 changed their sporting nationality after February 2022 (32). In total, Russian-born athletes won 17 medals at the 2024 Paris Olympics, including six gold, two silver, and nine bronze. Six of them changed their nationality after the start of the conflict: Akhmed Tazhudinov (Bahrain, freestyle wrestling, gold); Razambek Zhamalov (Uzbekistan, freestyle wrestling, gold); Ekaterina Antropova (Italy, volleyball, gold); Anastasia Kirpichnikova (France, swimming, silver); Dauren Kurugliev (Greece, freestyle wrestling, bronze); Chermen Valiev (Albania, freestyle wrestling, bronze) (49).

Again, this poses a number of sport integrity considerations. How did the migration of such strong athletes affect procedural (competition) integrity? Did it enrich the competition, by bringing more skill, or did it ruin the balance by strengthening particular countries, which did not invest effort and time in the long-term preparation of these star athletes? Does the acquisition of these athletes align with the organisational integrity of destination federations, as they do not only take the skilled leaders from Russian national teams but also get an instant advantage over other countries, who were represented by their “own” athletes. And should the skill of the athletes make any difference at all in this challenging discussion? Some of these questions concerning procedural integrity have already begun to attract academic debate, and further research, enriched by the newest geopolitical contexts, will help advance this critical discussion (50).

### 3.4 Popular destinations

If we look at the data in Table 3, it becomes clear that the most common destinations for Russian athletes post-2022 either coincide with the general migration patterns of Russians (e.g., Israel, Serbia, Armenia, Kazakhstan, Uzbekistan, Georgia, Turkey) or are historically leading sports countries (Germany, the United States, France, the United Kingdom, Spain). Looking at this through the classical pull and push factors migration theory (51), we can name a few main pull factors for Russian athletes, including cultural or ethnic ties, robust training infrastructure and funding, and personal connections.

**Table 3:** Destinations of Russian athletes who changed their sporting nationality after February 2022, by country.

Country	Athletes	Country	Athletes
Israel	29	Finland	9
Serbia	29	Georgia	8
Germany	23	Montenegro	8
USA	20	Canada	7
Armenia	19	Turkey	7
France	18	Hungary	7
Kazakhstan	16	Italy	7
United Kingdom	15	Kyrgyzstan	6
Poland	12	Palestine	6
Spain	12	Azerbaijan	6
Uzbekistan	10	Austria	5

Estimate of Novaya Gazeta, July 2024 (29).

For instance, athletes with non-Russian ethnic backgrounds often already hold a second citizenship or can acquire one more easily, thus simplifying the switch. Consequently, ethnic Armenians gravitate toward Armenia, ethnic Georgians toward Georgia, and Jewish athletes toward Israel. Even those without such backgrounds often find it more comfortable to adapt to other post-Soviet or Slavic states. Established sports powerhouses become a destination for many athletes because they may already reside there for training purposes and, in some cases, have already secured permanent residency or citizenship. Personal connections also play a significant role: three of the eight Albanian athletes at the 2024 Paris Olympics are former Russian wrestlers, likely due to the involvement of Russian coaches with the Albanian national team (46). Finally, some countries become destinations due to the less intense competition and subsequently easier qualification for world championships and Olympics.

### 3.5 Responses on athlete migration from stakeholders in Russia

The migration of athletes caused intense debates in Russia, both among professionals and in society. Although some radical voices claimed migrated athletes as traitors, public persons in sports and politics were softer in their estimates. For instance, Stanislav Pozdnyakov (President of the Russian Olympic Committee, 2018–2024) acknowledged that athletes have the right to change citizenship, as everyone is free in their decisions, but emphasised: “The best response to this is to provide better training conditions, competition opportunities, and social programs. Those who switched citizenship will soon realise their mistake” (52). Later in 2024, Russian Minister of Sport Mikhail Degtyarev expressed scepticism about athletes changing nationality, stating: “It is unfair that athletes competing under a neutral flag are being criticized—they did not renounce their flag. But as for those who change sporting nationality, I personally have questions, and I am sure most Russians do as well” (53). Such thoughts are supported by a Chairman of the State Duma Committee on Physical Culture and Sport Dmitry Svischev: “The state, along with parents, clubs, regions, and schools, has invested significant funds into developing athletes. To see a world champion or national team member simply taken away by a country offering a higher salary seems unfair” (54). These positions are due to the fact that most Russian sports schools are state-funded and youth training is largely free. In light of stakeholder integrity, it seems logical for officials to raise the issue of financial or other forms of compensation for the development of valuable athletes or appeal to patriotic loyalty.

Further, such appeals target the personal integrity of athletes, putting them in front of a difficult ethical choice: to save their careers or to acknowledge patriotic values first. The decisions of sport governing bodies literally put athletes in the corner: For example, two championing light-heavyweight professional boxers, Artur Beterbiev and Dmitry Bivol, who spent their entire career representing Russia, changed their sporting nationality but expressed unhappiness about it, explaining that otherwise they would not have a chance to compete. Bivol commented on his first fight under Kyrgyz flag: “I don't want to offend anyone, but I never even considered competing under the flag of Kyrgyzstan […] They wanted to cancel the fight because of this situation. They said I couldn't enter with the [Russian] flag, with the anthem, or even use Viktor Tsoi's music [Soviet rock idol], which I always walk out to […] those who support me know where I'm from, where I trained, and which country I represent.” (48). Beterbiev, an ethnic Chechen who has lived in Canada since 2013, expressed a similar opinion: “I have always represented the Russian flag, and entering the ring under the Canadian flag was a forced measure. They could have simply banned me and stripped me of my titles” (48). Again, this brings up the question of the whole national allegiance concept in sport and the need for its reconceptualization (or abolition), as individual human beings should not be forced to make such difficult decisions as nationality change just to keep doing their job. Putting athletes in such position raises questions regarding the organisational integrity of sport governing bodies as well as procedural integrity of competitions, which could have developed better mechanisms to cope with geopolitical crises.

Many renown retired athletes in Russia expressed understanding for decisions taken by athletes. For instance, Olympic tennis champion Evgeni Kafelnikov claimed: “An athlete's career is short—just like their biological resources. They cannot wait indefinitely for political changes that will bring Russia back into international sports. I cannot condemn them for making this decision. This trend will unfortunately continue, but I believe we have no right to judge these athletes” (55). Legendary figure skating coach Tatiana Tarasova agreed, stating: “Russian athletes will inevitably change nationality—there's no need to cling to this or condemn them. An athlete's career happens only once.” (54). Three-time Olympic figure skating champion Irina Rodnina adds another aspect to support the emigrant Russian athletes: “The most important thing is that these athletes continue to bring honour to their native people and their coaching school” (56).

Yet, the athlete community is not that unanimous. For example, the president of the Russian Figure Skating Federation, Olympic Champion Anton Sikharulidze distinguishes between different cases of nationality change: “If an athlete, either as an adult or with their family as a minor, relocates, gains citizenship, and integrates into their new country, that is their right. However, if nationality is changed simply to compete in events, I do not support it—it seems wild to me” (57). Such positions are understandable too: sport administrators are interested stakeholders in the situation, who do not want athletes from respective federations to leave.

These positions reflect the interests of different stakeholders involved in the case (state officials, sport administrators, and athletes), and illuminate the multi-layered structure of sports integrity. While the first group emphasises the investments made in athlete development and the need for compensation or patriotic loyalty, many former and current athletes argue that the decision is an individual necessity shaped by career longevity and opportunities. The discussion remains complex, with varying perspectives even within the sports community—some viewing it as an inevitable reality, while others seek to differentiate between legitimate relocation and opportunistic switches for competition purposes. The differing views among sports administrators, policymakers, and athletes suggest a complex web of motivations, requiring a nuanced examination of governance mechanisms, contractual obligations, and ethical frameworks in international sports migration.

## 4 Research agenda

This brief research report offers a unique perspective on athlete migration. The descriptions demonstrate the multi-layered complexity of athlete migration following geopolitical developments and substantiate the need for further, detailed research on stakeholder interests and responsibilities in athlete migration. Based on our case study, future research themes are outlined in the following to empirically examine the situation through the lens of sports governance, ethics and integrity:

### 4.1 Analysis of stakeholders’ interests, rights, and responsibilities

This research theme seeks to evaluate the roles of various parties involved in the migration of Russian athletes, including athletes themselves, international sport governing bodies, and Russian sport organisations. Exploring this topic, it seems logical to propose a study that combines qualitative analysis of the data gathered through semi-structured interviews with various stakeholders. Grey literature, periodicals, and documents from official sources (e.g., websites of sport governing bodies) can complement the data. The research should aim to uncover the interrelations between these actors, revealing the complexities of their roles in the migration process and the influence of political and social factors.

### 4.2 Analysis of motivations and perceptions of key stakeholders involved in the migration of Russian athletes

This research theme seeks to uncover deeper insights into the values, conflicts, and decision-making processes of athletes and organisations involved in the migration process through empirical methods. Understanding these motivations is crucial to gaining a comprehensive view of athlete decision-making in politically charged environments, especially in light of the 2022 international sanctions. Investigations of the issue will contribute to the broader discourse on sports sociology, migration, and national identity, offering a human rights perspective through personal narratives.

### 4.3 In-depth ethics and integrity analysis of the Russian athletes’ case

Given the involvement of multiple stakeholders in the sporting nationality change process, it is essential to assess the ethical implications and examine how various dimensions of sport integrity are addressed in this case. These dimensions include personal, organisational, procedural, stakeholder and inherent integrity (7). This analysis will highlight broader ethical and governance challenges within the sports industry, thus laying the foundation for subsequent policymaking.

### 4.4 Comparative analysis of athlete migration in different geopolitical contexts

This research theme seeks to place the migration of Russian athletes within a broader comparative framework by analyzing historical cases of athlete migration driven by geopolitical crises. By examining cases such as the migration of Yugoslav athletes during the 1990s, and the movement of athletes from South Africa during the apartheid-related sanctions, such a study would provide critical insights into the similarities and differences across contexts. Utilizing comparative case study methodology, the research can highlight patterns in governance responses, athlete agency, and the role of international sport governing bodies in regulating migration during times of political instability, thus following their evolution and outlining the takeaways for policymaking.

To sum up, this brief report described the case of migration of Russian athletes after 2022. The uniqueness of the topic and its broader implications makes it an important direction for research, which should consequently help both policymakers, athletes, and fellow-academics.

## Data Availability

The original contributions presented in the study are included in the article/Supplementary Material, further inquiries can be directed to the corresponding author.

## References

[1] Maguire J Liston K Falcous M. Handbook on Sport and Migration. Cheltenham: Edward Elgar Publishing (2024).

[2] Babar Z Reiche D. From imported athletes to home-grown talents: long-term residents in qatari national sports teams. In: Maguire J Liston K Falcous M, editors. Handbook on Sport and Migration. Cheltenham: Edward Elgar Publishing (2024). p. 187–96.

[3] Maguire JA. ‘Real politic’ or ‘ethically based’: sport, globalization, migration and nation-state policies. Sport in Society. (2011) 14(7–8):1040–55. 10.1080/17430437.2011.603557

[4] Riordan J. Sport after the cold war: implications for Russia and Eastern Europe. In: Wagg S Andrews D, editors. East Plays West. London: Routledge (2006). p. 272–88.

[5] Exner J. Sporting Nationality in the Context of European Union Law: Seeking a Balance Between Sporting Bodies’ Interests and Athletes’ Rights. Cham: Springer (2019).

[6] Storey D. National allegiance and sporting citizenship: identity choices of ‘African’ footballers. Sport Soc. (2020) 23(1):129–41. 10.1080/17430437.2018.1555228

[7] Gardiner S Parry J Robinson S. Integrity and the corruption debate in sport: where is the integrity? Eur Sport Manag Quart. (2017) 17(1):6–23. 10.1080/16184742.2016.1259246

[8] Manoli AE Konstantopoulos I. Integrity and Sustainability in Sport: Business, Environmental and Social Goals. London: Taylor & Francis (2024).

[9] Syropoulos C Felbermayr G Kirilakha A Yalcin E Yotov YV. The global sanctions database–release 3: COVID-19, Russia, and multilateral sanctions. Rev Int Econ. (2024) 32(1):12–48. 10.1111/roie.12691

[10] BBC. Novye Rossiyskie Emigranty. Kto oni, Skol'ko ikh I Kuda Uekhali? [New Russian Emigrants. who are They, how Many, and Where did They go?] (2023). Available online at: https://www.bbc.com/russian/features-65686712 (Accessed March 30, 2025)

[11] Forbes. Privychnyi Kvest: Kuda I Pochemu Vnov’ Pereezzhali v 2024 Godu Rossiiskie Relokanty [Familiar Quest: Where and why Russian Relocants Moved Again in 2024] (2024). Available online at: https://www.forbes.ru/svoi-biznes/527913-privycnyj-kvest-kuda-i-pocemu-vnov-pereezzali-v-2024-godu-rossijskie-relokanty (Accessed March 30, 2025)

[12] NEWHR. Relokatsiya IT Spetsialistov [Relocation of IT Specialists] (2024). Available online at: https://newhr.org/data/itrelocation22-24 (Accessed March 30, 2025).

[13] Kazantseva NV Ostapenko VA. Relokaciya specialistov iz Rossii: masshtaby i ekonomicheskie posledstviya [relocation of specialists from Russia: scale and economic consequences]. Upravlenie Personal Intellekt Resursami Rossii. (2023) 12(2):24–9. 10.12737/2305-7807-2023-12-2-24-29

[14] Vedomosti. RANHiGS: V Rossiyu Vernulis’ 10% Relokantov [RANEPA: 10% of Relocants Came Back to Russia] (2025). Available online at: https://www.vedomosti.ru/society/articles/2025/02/07/1090699-v-rossiyu-vernulis (Accessed March 30, 2025)

[15] Altukhov S. Governance dysfunction in world sport: issues raised by the conflict in Ukraine. In: Chadwick S Widdop P Goldman M, editors. The Geopolitical Economy of Sport. London: Routledge (2023). p. 36–41.

[16] Schubert M. Neutrality of the Olympic movement against recent developments in sport and geopolitics–need of reconceptualisation. Int Sports L J. (2024) 24(2):143–61. 10.1007/s40318-024-00281-w

[17] Jamali A Kozlová A Whelan KA Faix M. Game over for Russian athletes? Human rights aspects of measures adopted by international sports organisations as a response to the Russian aggression against Ukraine. Int Comp L Rev. (2023) 23(1):182–209. 10.2478/iclr-2023-0008

[18] Næss HE. Sport governing bodies and the prioritization of human rights: a conceptual analysis of the international Olympic committee’s (IOC) dispute with Russia. Sport Ethics Phil. (2025) 19(2):142–55. 10.1080/17511321.2024.2317127

[19] Absalyamov T Schubert M. How the Ukraine conflict affects Russian football clubs: the hostages of big politics. In: Chadwick S Widdop P Goldman M, editors. The Geopolitical Economy of Football: Where Power Meets Politics and Business. London: Routledge (2024). p. 66–76.

[20] Gulina O. Emigration from Russia after 24 February 2022: main patterns and developments. Migration Observervatory (2023). p. 64.

[21] Kamalov E Sergeeva I Zavadskaya M Kostenko V. Six Months in exile: A new Life of Russian Emigrants. European University Institute (2023).

[22] Karaçay AB. The “new wave” of Russian emigration to Türkiye. Int Rel Int L J. (2023) 104(4). 10.26577/IRILJ.2023.v104.i4.06

[23] Melkumyan Y Melkonyan N. Immigration of Russian citizens to Armenia during the Russian-Ukrainian war that began in 2022: pull-push factors. J Political Sci. (2023) 2(1(4)):137–47. 10.46991/JOPS/2023.2.4.137

[24] Ortiz D Myers D Walls E Diaz ME. Where do we stand with newspaper data? Mobilization. (2005) 10(3):397–419. 10.17813/maiq.10.3.8360r760k3277t42

[25] Goretti L. The Sporting Sanctions Against Russia: Debunking the Myth of Sport’s Neutrality. Istituto Affari Internazionali (IAI) (2022).

[26] Sport-Express. “Eto Diskriminatsiya po Natsionalnomu Priznaku”. ATP Vstupilas za Rossiyan Posle Izgnaniya s Uimbldona. [“This is Discrimination by Nationality”. ATP Backs Russians After Wimbledon’s ban] (2022). Available online at: https://www.sport-express.ru/tennis/grand-slam/reviews/tennis-reakciya-atp-tura-v-svyazi-s-zapretom-rossiyskim-igrokam-vystupat-na-uimbldonskom-turnire-1917456/ (Accessed March 30, 2025)

[27] International Olympic Committee. Q&A Regarding the Participation of Athletes with a Russian or Belarusian Passport in International Competitions (2023). Available online at: https://www.olympics.com/ioc/media/q-a-on-solidarity-with-ukraine-sanctions-against-russia-and-belarus-and-the-status-of-athletes-from-these-countries (Accessed March 30, 2025)

[28] RIA Novosti. Sikharulidze: Nekotorye Figuristy, Smenivshie Grazhdanstvo, Prosyatsya Obratno [Sikharulidze: Some Figure Skaters who Changed Citizenship are Asking to Return] (2023). Available online at: https://rsport.ria.ru/20230906/figuristy-1894595938.html (Accessed March 30, 2025)

[29] Novaya Gazeta Europe. Emigrantskii Zachet [Emigration Score] (2024). Available online at: https://novayagazeta.eu/articles/2024/07/26/emigrantskii-zachet (Accessed March 30, 2025)

[30] Fédération Internationale des Échecs. FIDE resolution on the Russian Federation Joining the ACF (2023). Available online at: https://www.fide.com/news/2247 (Accessed March 30, 2025)

[31] Sport-Express. Za Mesyats Bol’she 10 Nashikh Figuristov Reshilis’ smenit’ Sbornuyu [in a Month, More Than 10 of our Figure Skaters Decided to Change Their National Team.] (2024). Available online at: https://www.sport-express.ru/figure-skating/reviews/kto-iz-rossiyskih-figuristov-smenil-sportivnoe-grazhdanstvo-v-2024-godu-samodelkina-morozov-titova-geynish-i-chigirev-2218032/ (Accessed March 30, 2025)

[32] Rossiyskaya Gazeta. Pochemu Rossiiskie Sportsmeny Menyayut Grazhdanstvo [why Russian Athletes Change Citizenship] (2024). Available online at: https://rg.ru/2024/10/11/porvat-s-flagom.html (Accessed March 30, 2025)

[33] Sport-Express. Zvezdy Konnogo Sporta Rossii Odin za Drugim Berut Drugoe Sportivnoe Grazhdanstvo [the Stars of Russian Equestrian Sports are Taking Another Sports Citizenship one After Another] (2023). Available online at: https://www.sport-express.ru/equestrian/reviews/pochemu-sportsmeny-sbornoy-rossii-po-konnomu-sportu-massovo-menyayu-grazhdanstvo-iz-za-sankciy-2036547/ (Accessed March 30, 2025)

[34] TASS. V Sbornoy Rossii po Konnomu Sportu Nazvali Prichinu Perekhoda Tuganova v Komandu Palestiny [the Russian Equestrian Team Named the Reason for Tuganov’s Transfer to the Palestinian Team] (2023). Available online at: https://tass.ru/sport/17030251 (Accessed March 30, 2025)

[35] Championat. Sbornaya Rossii Teryaet Igrokov. Kto Uzhe Smenil Sportivnoe Grazhdanstvo? [Team Russia Loses Players. who has Already Changed Sporting Nationality?] (2023). Available online at: https://www.championat.com/football/article-5228485-rossijskie-futbolisty-smenivshie-grazhdanstvo-tiknizyan-sevikyan-sazonov-maradishvili-zuev.html (Accessed March 30, 2025)

[36] Match TV. Tiknizyan Zayavil, Chto Otstranenie Sbornoy Rossii Povliyalo na Yego Vybor v Pol'zu Armenii [Tiknizyan Claims Russia’s ban Influenced his Choice for Armenia] (2023). Available online at: https://matchtv.ru/football/matchtvnews_NI1841617_Tiknizan_zajavil_chto_otstranenije_sbornoj_Rossii_povlijalo_na_jego_vybor_v_polzu_Armenii (Accessed March 30, 2025).

[37] Gleaves J Llewellyn M. Ethics, nationalism, and the imagined community: the case against inter-national sport. J Phil Sport. (2014) 41(1):1–9. 10.1080/00948705.2013.785427

[38] Iorwerth H Hardman A. The case for inter-national sport: a reply to Gleaves and Llewellyn. J Phil Sport. (2015) 42(3):425–41. 10.1080/00948705.2015.1036876

[39] Maguire J. Blade runners: Canadian migrants, ice hockey, and the global sports process. J Sport Soc Iss. (1996) 20(3):335–60. 10.1177/019372396020003007

[40] Agergaard S Botelho VL Tiesler NC. The typology of athletic migrants revisited: transnational settlers, sojourners and mobiles. In: Agergaard S Tiesler N, editors. Women, Soccer and Transnational Migration. London: Routledge (2014). p. 191–215.

[41] Magee J Sugden J. “The world at their feet” professional football and international labor migration. J Sport Soc Iss. (2002) 26(4):421–37. 10.1177/0193732502238257

[42] Championat. Shakhmatistka Aleksandra Kosteniuk Prokommentirovala Perekhod pod Flag Shveytsarii [Chess Player Aleksandra Kosteniuk Commented Transfer to Swiss Flag] (2023). Available online at: https://www.championat.com/other/news-5018053-aleksandra-kostenyuk-nazvala-prichinu-perehodu-pod-flag-sbornoj-shvejcarii.html (Accessed March 30, 2025)

[43] Championat. “Gorzhus’, Chto Budu Predstavlyat’ Frantsiyu”. Eshchyo Odna Chempionka Pokidaet Sbornuyu Rossii [“I'm Proud to Represent France”. Another Champion Leaves the Russian National Team.] (2023). Available at: https://www.championat.com/other/article-5088599-rossijskaya-plovchiha-anastasiya-kirpichnikova-menyaet-sportivnoe-grazhdanstvo-ona-budet-vystupat-za-sbornuyu-francii.html (Accessed March 30, 2025).

[44] Kommersant. Rossiyskaya Short-trekistka Prosvirnova Budet Vystupat’ za Sbornuyu Danii [Russian Short-track skater Prosvirnova Will Compete for the Danish National Team] (2023). Available online at: https://www.kommersant.ru/doc/5914130 (Accessed March 30, 2025)

[45] Championat. Vitse-chempionka OI-2020 iz Rossii Khochet Vystupat’ za Uzbekistan. Yeyo Nado Ponyat’ I Otpustit’ [the Silver Medalist of the Tokyo 2020 Olympics from Russia Wants to Compete for Uzbekistan. She Must be Understood and let go] (2023). Available online at: https://www.championat.com/other/article-5079417-grebchiha-anna-prakaten-perejdyot-iz-sbornoj-rossii-v-uzbekistan-pochemu-reshenie-vice-chempionki-olimpiady-mozhno-ponyat.html (Accessed March 30, 2025)

[46] Sport24. Na Olimpiade-2024 Budut Desyatki Sportsmenov iz Rossii. Kto oni I Pochemu Vystupayut za Drugie Strany? [There Will be Dozens of Athletes from Russia at the 2024 Olympics. who are They and why are They Competing for Other Countries?] (2024). Available online at: https://sport24.ru/other/article-665878-rossiyskiye-sportsmeny-na-olimpiyskikh-igrakh-2024-goda-v-parizhe-smena-sportivnogo-grazhdanstva-emigratsiya-foto-biografii (Accessed March 30, 2025)

[47] RIA Novosti. Rossiyskiy Borets Smenil Grazhdanstvo na Grecheskoe [the Russian Wrestler Changed his Citizenship to Greek] (2023). Available online at: https://rsport.ria.ru/20230416/kurugliev-1865654822.html (Accessed March 30, 2025)

[48] Sports”. Dva Glavnykh Rossiyskikh Boksory Poderyutsya ne pod Flagom Rossii. Kak tak? [Two top Russian Boxers Will Fight not Under the Russian Flag. how come?] (2024). Available online at: https://www.sports.ru/boxing/blogs/3270034.html (Accessed March 30, 2025)

[49] Sport RBC. Kto iz Smenivshikh Grazhdanstvo Rossiyan Zavoyeval Medali na Olimpiade-2024 [who among the Russians who Changed Their Citizenship won Medals at the 2024 Olympics?] (2024). Available online at: https://sportrbc.ru/news/66b9a5069a7947182f27a862 (Accessed March 30, 2025)

[50] Phan J. Foreign talent, local glory: can national excellence be outsourced? Sport Ethics Phil. (2013) 7(2):186–201. 10.1080/17511321.2013.779312

[51] Lee ES. A theory of migration. Demography. (1966) 3:47–57. 10.2307/2060063

[52] Sport RBC. Glava OKR Zayavil, Chto Smenivshie Grazhdanstvo Sportsmeny Poymyut Svoyu Oshibku [the Head of the Russian Olympic Committee Stated That Athletes who Changed Their Citizenship Will Realize Their Mistake] (2023). Available online at: https://sportrbc.ru/news/650eb33a9a7947738349ed40?from=copy (Accessed March 30, 2025)

[53] TASS. Degtyarev Uveren, Chto Smena Rossiyskimi Atletami Grazhdanstva Vyzyvayet Voprosy u Grazhdan RF [Degtyarev is Confident That the Change of Citizenship by Russian Athletes Raises Questions among Russian Citizens] (2024). Available online at: https://tass.ru/sport/21763369 (Accessed March 30, 2025)

[54] Sport-Express. Minsport Rasskazal ob Otyezde iz Rossii 67 Sportsmenov. Mnogoe eto ili Malo [the Ministry of Sport Spoke About the Departure of 67 Athletes from Russia. is This a lot or a Little?] (2023). Available online at: https://www.sport-express.ru/obshchestvo/reviews/kak-mnogo-sportsmenov-iz-rossii-menyayut-sportivnoe-grazhdanstvo-oficialnye-dannye-ministerstva-sporta-2111397/ (Accessed March 30, 2025)

[55] Gazeta.ru. “Sportsmeny ne Mogut Zhdat’ u Morya Pogody": 55 Atletov Smenili Grazhdanstvo Radi Olimpiady [“Athletes Can’t Wait for a Change in Circumstances”: 55 Athletes Changed Their Citizenship for the Olympics] (2023). Available online at: https://www.gazeta.ru/sport/2023/09/07/17547878.shtml (Accessed March 30, 2025)

[56] Lenta.ru. “Ikh Postupok Vyzyvayet Voprosy”. Rossiyskiy Minister Raskritikoval Smenivshikh Grazhdanstvo Sportsmenov [“Their Actions Raise Questions.” the Russian Minister Criticized Athletes who Changed Citizenship] (2024). Available online at: https://lenta.ru/news/2024/09/04/ih-postupok-vyzyvaet-voprosy-rossiyskiy-ministr-raskritikoval-smenivshih-grazhdanstvo-sportsmenov/ (Accessed March 30, 2025)

[57] TASS. Sikharulidze Schitayet “dikovatoy” Smenu Grazhdanstva Tol'ko Radi Uchastiya v Sorevnovaniyakh [Sikharulidze Considers Changing Citizenship Solely for Participation in Competitions to be “wild”] (2025). Available online at: https://tass.ru/sport/23194569 (Accessed March 30, 2025)

